# The Relationship Between Bone Mineral Density and Body Composition Among Qatari Women With High Rate of Obesity: Qatar Biobank Data

**DOI:** 10.3389/fnut.2022.834007

**Published:** 2022-04-05

**Authors:** Abdelhamid Kerkadi, Shalima Lathief, Yasmen Khial, Toka Teleb, Grace Attieh, Md Mizanur Rahman, Zumin Shi, Abdelali Agouni

**Affiliations:** ^1^Department of Human Nutrition, College of Health Sciences, QU-Health, Qatar University, Doha, Qatar; ^2^Biological Science Program, Department of Biological and Environmental Sciences, College of Arts and Sciences, Qatar University, Doha, Qatar; ^3^Department of Pharmaceutical Sciences, College of Pharmacy, QU-Health, Qatar University, Doha, Qatar

**Keywords:** bone mineral density, body component composition, Qatar, women, abdominal obesity

## Abstract

Studies have reported inconsistent results for the relationship between body composition and bone mineral density (BMD) among women, especially those with a high rate of obesity. This study aims to examine the association between BMD and body composition among Qatari women. A cross-sectional study, using data from the Qatar Biobank (QBB), was conducted on 2,000 Qatari women aged 18 and over. Measurements were taken by dual-energy X-ray absorptiometry (DEXA) for body composition [visceral fat and android fat (AF)], gynoid fat (GF), trunk fat, total fat mass (TFM), total lean mass (LM) and bone mineral density (BMD), including the lumber spine, neck, femur and total body. The participants were divided into groups of normal and low BMD, based on their T-score. Non-linear regression analysis using the restricted cubic spline method was performed according to the T-score of the total BMD for the fat mass variables. Women with a low BMD (T-score <-1) had significantly lower body composition indicators. LM was positively correlated with BMD at the spine (*r* = 0.29, *p* < 0.001), neck (*r* = 0.32, *p* < 0.001), and femur (*r* = 0.28, *p* < 0.001), as well as total BMD (*r* = 0.29, *p* < 0.001) and T-score (*r* = 0.31, *p* < 0.001), while the correlatio between TFM and BMD was negative and weak (*r* = −0.05, <0.017). Results of the non-linear regression indicated that components of fat distribution (TFM, AF, GF and trunk fat) were positively associated with total body T-score. In the adjusted non-liner regression, only a slight increase in T-score was recorded with an increase in FM. The association between FM and BMD was non-linear, suggesting that FM may not be a strong protector of bones among women with high rate of obesity.

## Introduction

Bone mineral density (BMD) is the amount of bone mineral content that is measured by dual-energy x-ray absorptiometry (DEXA) in g/cm^2^ and is used in the diagnosis of osteoporosis (1, 2). Many factors are known to affect BMD, such as age, sex, vitamin D status (2, 3), the use of certain medications (e.g., glucocorticoids) (4) and obesity (5). Long-term vitamin D deficiency causes a decline in BMD, as a decrease in the levels of vitamin D affects an adequate absorption of calcium. Therefore, bone demineralisation occurs to maintain serum calcium homeostasis (3). According to the 2016–2017 annual report of the Qatar Biobank (QBB) (6), almost 86% of the study population aged 18–65 years, were deficient in vitamin D. The deficiency was found to be higher in women (65%) compared with men (35%), while 70% were obese.

Obesity is one of the modifiable factors associated with BMD (7). Globally, obesity and osteoporosis are both health problems that are closely related to morbidity and mortality (8). Both conditions have been reported due to impaired regulation of the same bone marrow mesenchymal stromal cell (9). In 2016, the prevalence of obesity was higher among women (43.2%) than men (39.5%) in Qatar (10). According to Bener et al. (11), the prevalence of osteoporosis was higher (12.3%) among postmenopausal Qatari women with a high body mass index (BMI) compared with premenopausal women. The association between obesity and BMD is controversial and has not been fully explained (12). Obesity has been reported to be positively associated with BMD (13–15), probably due to the mechanical effect of increased body weight on bone (16), as well as the higher levels of adipose-derived hormones, such as leptin, that promote bone growth (17). On the other hand, some studies have shown that obesity may have a negative effect on BMD (18, 19).

Regarding obesity, it is necessary to consider lean mass (LM) and fat mass (FM), as both of these components of body composition are found to be associated with BMD (20). Several studies have attempted to explain their association with BMD (21–28), although research has provided contradicting findings on the contribution of LM and FM to changes in BMD. Some studies have asserted a positive association between LM and BMD (27, 29), while others (17) found that LM, rather than FM, was the strongest predictor of BMD. In contrast, some studies showed that FM (30) and android /gynoid fat mass ratio (A/G FMR) were positively associated with BMD (22, 31). Nevertheless, other studies reported that both FM and LM have a positive association with BMD (32), while some have observed that the association of FM became negative after adjusting for BMI (15) and weight (23). Additionally, other research has found that FM is negatively correlated to BMD in premenopausal women and positively correlated in postmenopausal women (23, 33).

In view of these inconsistent findings, and the absence of a study in Qatar, this study aims to examine the association between BMD and body composition among Qatari women using data from the QBB.

## Methods

### Brief Summary of Qatar Biobank Survey

The QBB is considered to be the first population-based prospective cohort study. Established in 2012, it is partnered with the Ministry of Public Health (MOPH) and the Hamad Medical Corporation (HMC). The QBB continuously gathers biological samples and collects information about the lifestyle and health status of nationals and residents (34).

### Study Population

This study included 2,000 samples of Qatari women aged 18 and over, which were selected from the master database of the QBB. Samples were chosen by using a simple random selection method in which each sample had an equal chance of being selected. Excluded samples comprised those from males, pregnant and breastfeeding women and women with chronic diseases, such as hypertension, diabetes mellitus, cancer and chronic kidney disease. Also excluded were women with endocrine diseases (Cushing syndrome, heavy or irregular menses), those following a restrictive diet to lose weight, and women who were using medical treatments with growth hormones. Among the total sample, eight participants were excluded for data missing. The analysis was performed on 1992 participants.

Ethical approval was obtained from the institutional review board of the QBB (EX-2021-QF-QBB-RES-ACC-00040-0166) and was carried out according to the Declaration of Helsinki. All participants in this study provided their written consent to share all their data in research studies, using an identification number but without revealing their identity.

### Obesity Indicators, Anthropometric and DXA Derived Parameters

A trained healthcare team at the QBB used standard methods to record the anthropometric measurements. The participants were asked to wear light clothes, without shoes, when measurements were being taking for height and weight. The measurements included those for weight, height, waist circumference (WC) and hip circumference (HC).

Body weight (kg) and height (cm) were measured by using a calibrated scale and a wall-mounted stadiometer (Seca, Hamburg, Germany). Using a non-stretchable tape, WC (cm) was determined at the abdominal region, at the level of the umbilicus at the midpoint between the last rib of the body and the top of the iliac crest. Waist to hip ratio (WHR) was also calculated. Overall adiposity, total body fat (TBF), visceral fat, and regional fat distribution (trunk, AF and GF) and BMD were measured using Lunar iDXA (SN 210520, GE Healthcare, USA). For BMD, three regions of interest were measured on the GE Lunar iDXA machine—the anteroposterior (AP) lumbar spine, the left femur and the whole-body composition. The Lunar iDXA performed a daily six-point calibration which provided highly sensitive measurements with normal, osteopenic and osteoporotic BMD values, as well as lean, normal and obese values (34).

Central obesity was determined by using WC, defined as WC ≥102 cm for men, and WC ≥88 cm for women. BMI was calculated using weight (kg) over the square height in meters (m) (35). Therefore, overweight (OW) was defined as BMI = 25–29.9 kg/m^2^ and obesity (OB1) as BMI = 30–34.9 kg/m^2^; (OB2) as BMI= 35–39.9 kg/m^2^ and (OB3) BMI >40 kg/m^2^. Measurements were obtained by Lunar iDXA (SN 210520, GE Healthcare, USA) BMD indicators in the lumbar spine, femoral neck (FN), femur and total BMD, and the total body T-score (Difference in the BMD between participant and a healthy young adult). The results of the BMD measurements were expressed in g/cm^2^.

### Covariates

Self-administered health and lifestyle questionnaires were used to obtain data such as age, level of education, smoking status and physical activity. The level of education was divided into three categories—lower education, up to secondary school; medium, technical or professional school; and higher education, University and above. The physical activity levels were expressed as metabolic equivalents (METs) in hours per week were calculated based on the frequency and duration of the different types of physical activity Face-to-face interviews were conducted at the QBB clinic by professional nurses, to gather information about health status, related family medical history and usage of medications of participants.

### Statistical Analysis

The data was analyzed using SPSS for Windows (version 23) and STATA (version 17), and the results were presented as mean and standard deviations (SD) for continuous variables and as percentages for categorical variables. The participants were categorized into two groups, normal and low BMD, based on the z-score for total body BMD (T-score >-0.99, normal BMD; T-score <-1, low BMD) as per criteria from the World Health Organization (36). A comparison between the groups was made using a *t*-test for continuous variables and a chi-square test (χ^2^) for categorical variables. The non-linear association between different types of FM indicators and the T-score of total BMD was assessed by the restricted cubic spline method, with models adjusted for age, smoking, physical activity and the use of supplements. Three knots were put at the 10, 50, and 90th percentile. A *p*-value for non-linearity was obtained by testing the regression coefficient of the second spline equal to zero, while the overall *p*-value for the association was obtained by testing the regression coefficient of the two splines simultaneously equal to zero. The results were visually presented, and the statistical significance for analysis was detected at *p* < 0.05.

## Results

### Sample Characteristics

Characteristics of the participants categorized according to their z-scores of the total BMD are presented in Table 1. The mean age of the participants was 51.55 ± 8.01 years. Compared with participants in the low BMD category, those in the normal BMD category were younger (*p* < 0.001). Most of the participants had obtained higher education (40.1%), and there was no significant difference in levels of education between those in the normal and low BMD groups. More than half of the participants were using vitamin supplements (51.1%), and there was no significant difference in the number of vitamin supplement users among the two categories.

**Table 1:** Characteristics of the study population according to BMD category^*a*^.

**Demographics**	**Normal BMD** **(z-score >-0.99)** ***n* = 1,649**	**Low BMD** **(z-score <-1)** ***n* = 343**	**Total** ***n* = 1992**
Age, years	50.91 ± 7.6	54.52^***^± 9.16	51.6 ± 8.0
**Education level**, ***n*** **(%)**^***b***^
Lower education^*c*^	628 (38.1)	144 (42.0)	772 (38.7)
Medium education^*d*^	357 (21.6)	65 (19.0)	422 (21.2)
Higher education^*e*^	664 (40.3)	134 (39.1)	798 (40.1)
Vitamin Supplement use, *n* (%)^*f*^	508 (51.3)	104 (50.2)	612 (51.1)

a *Values are expressed as mean ± SD for continuous variables and n (%) for categorical variables. Independent t-tests were used for continuous variable and λ^2^ tests for categorical variables*.

*Significant P-values are indicated by*

*** *p < 0.001. BMD, bone mineral density*.

b *λ^2^ = 2.192, p = 0.334*.

c *Education up to secondary school*.

d *Technical or professional school*.

e *University and above*.

f *λ^2^ = 0.288, p = 0.866*.

### Anthropometric Measurements

The anthropometric measurements according to BMD category are shown in Table 2. Participants with a normal BMD were heavier and had a high BMI, WC and HC. There was a significant difference between those with normal and low BMD in body composition indicators (visceral FM, android FM, gynoid FM, trunk FM, total FM and total LM), with high values among the normal BMD categories. Those who were OW accounted for 38.8% of the participants, with a high prevalence among those with a low BMD (47.1%). More than half of the participants were obese (51.5%), and the prevalence of obesity 1, 2 and 3 was 10.3, 27.1, and 14.1%, respectively. There was a significant difference in the prevalence of obesity 2 and obesity 3 among the participants, with a higher proportion among the normal BMD category, 29.9 and 16.1%, respectively. However, there was no significant difference in the prevalence of obesity 1 among those with a normal BMD (10.3%) and low BMD (10.2%).

**Table 2:** Anthropometric measurements according to BMD category^*a*^.

**Anthropometric measurements**	**Normal BMD (z-score >-0.99)** ***n* = 1,649**	**Low BMD (z-score <-1)** ***n* = 343**	**Total** ***n* = 1,992**
Height (cm)	156.72^***^± 5.76	154.70± 6.29	156.37 ± 5.90
Weight (Kg)	81.23^***^± 14.34	71.54 ± 14.78	79.56 ± 14.89
BMI (Kg/m^2^)	33.10^***^± 5.73	29.90 ± 5.86	32.55 ± 5.87
WC (cm)	92.61^***^± 11.71	87.33 ± 12.41	91.69 ± 11.99
HC (cm)	112.44^***^± 11.04	106.95± 11.65	111.49 ± 11.33
WHR	0.82 ± 0.08	0.82 ± 0.09	0.82 ± 0.08
Visceral FM (g)	1,075.67^***^± 530.55	884.89 ± 491.31	1,042.81 ± 528.84
Android FM (g)	3,170.78^***^± 1,064.49	2,643.77 ± 1,015.79	3,080.63 ± 1,074.57
Gynoid FM (g)	6,219.69^***^± 1,663.11	5,434.56 ± 1,539.08	6,085.39 ± 1,668.55
Trunk FM (g)	19,129.25^***^± 5,715.36	16,099.39 ± 5,377.09	18,610.98 ± 5,771.55
Total FM (g)	38,070.24^^***^^± 9,921.97	32,866.83 ± 9,544.59	37,180.18 ± 10,049.04
Total LM (g)	40,370.52^***^± 5,388.08	35,949.42 ± 5,068.78	39,614.28 ± 5,587.48
**BMI categories**, ***n*** **(%)**^***b***^			
UW	0 (0)	2 (0.6)	2 (0.1)
NW	81 (4.9)	58 (16.9)	139 (7)
OW	436 (26.4)	130 (37.9)	566 (28.4)
OB 1	593 (36)	95 (27.7)	688 (34.5)
OB 2	351 (21.3)	42 (12.2)	393 (19.7)
OB 3	188 (11.4)	16 (4.7)	204 (10.2)

a *Values are expressed as mean ± SD for continuous variables and n (%) for categorical variables. Independent t-tests were used for continuous variable and λ^2^ tests for categorical variables*.

*Significant P-values are indicated by*

*** *p < 0.001. WC, Waist circumference; HC, hip circumference; WHR, waist-to-hip ratio; FM, Fat mass; LM, Lean mass; UW, Underweight; NW, Normal weight; OW, Overweight; OB 1, Obese type 1; OB 2, Obese type 2; OB 3, Obese type 3*.

b *λ^2^ = 111.3, p < 0.01*.

### Association Between Bone Mineral Indicators and Body Composition Indicators

The partial correlation between indicators for bone mineral and body composition is shown in Table 3. Visceral FM (0.09; *p* < 0.001) and trunk FM (0.06; *p* = 0.009) were positively correlated with spine BMD. On the other hand, gynoid FM (−0.06; *p* = 0.008) was negatively correlated with spine BMD. All FM variables did not show a significant association with femoral neck (FN) BMD. A significant negative correlation with femur BMD was observed for gynoid FM (−0.13; *p* < 0.001) and total FM (−0.10; *p* < 0.001). In contrast, visceral FM (0.08; *p* < 0.001) showed a positive correlation with femur BMD. Additionally, android FM (−0.05; *p* = 0.038) and total FM (−0.05; *p* = 0.017) were negatively associated with total BMD. A negative correlation was found between total BMD T-score and gynoid FM (−0.10; *p* < 0.001) and total FM (−0.08; *p* < 0.001). Compared with other body composition variables, total LM had a significantly higher positive association with all bone mineral indicators.

**Table 3:** Partial correlation between bone mineral indicators and body composition variables^*a*^.

	**Visceral FM (g)**		**Android FM (g)**		**Gynoid FM (g)**		**Trunk FM (g)**		**Total FM (g)**		**Total LM (g)**	
**Bone mineral indicators**	***r***	***p*-value**	***r***	***p*-value**	***r***	***p*-value**	***r***	***p*-value**	***r***	***p*-value**	***r***	***p*-value**
Spine BMD (g/cm^2^)	0.09	**<0.001**	0.01	0.595	−0.06	**0.008**	0.06	**0.009**	−0.02	0.285	0.29	**<0.001**
FN BMD^*b*^ (g/cm^2^)	0.03	0.168	−0.003	0.885	−0.003	0.906	0.03	0.130	0.02	0.412	0.32	**<0.001**
Femur^*b*^ BMD (g/cm^2^)	0.08	**<0.001**	−0.002	0.913	−0.13	**<0.001**	0.03	0.214	−0.10	**<0.001**	0.28	**<0.001**
Total BMD (g/cm^2^)	−0.02	0.345	−0.05	**0.038**	−0.03	0.120	−0.01	0.691	−0.05	**0.017**	0.29	**<0.001**
T-Score^*c*^	0.04	0.098	−0.02	0.456	−0.10	**<0.001**	0.02	0.446	−0.08	**<0.001**	0.31	**<0.001**

a *The correlation model was adjusted for age and BMI. r = correlation coefficient. Bold indicates statistically significant results. FN, Femoral neck*.

b *Left femur was measured*.

c *T-score of total BMD*.

### Association Between FM Variables and T-Score

**Figure 1:**
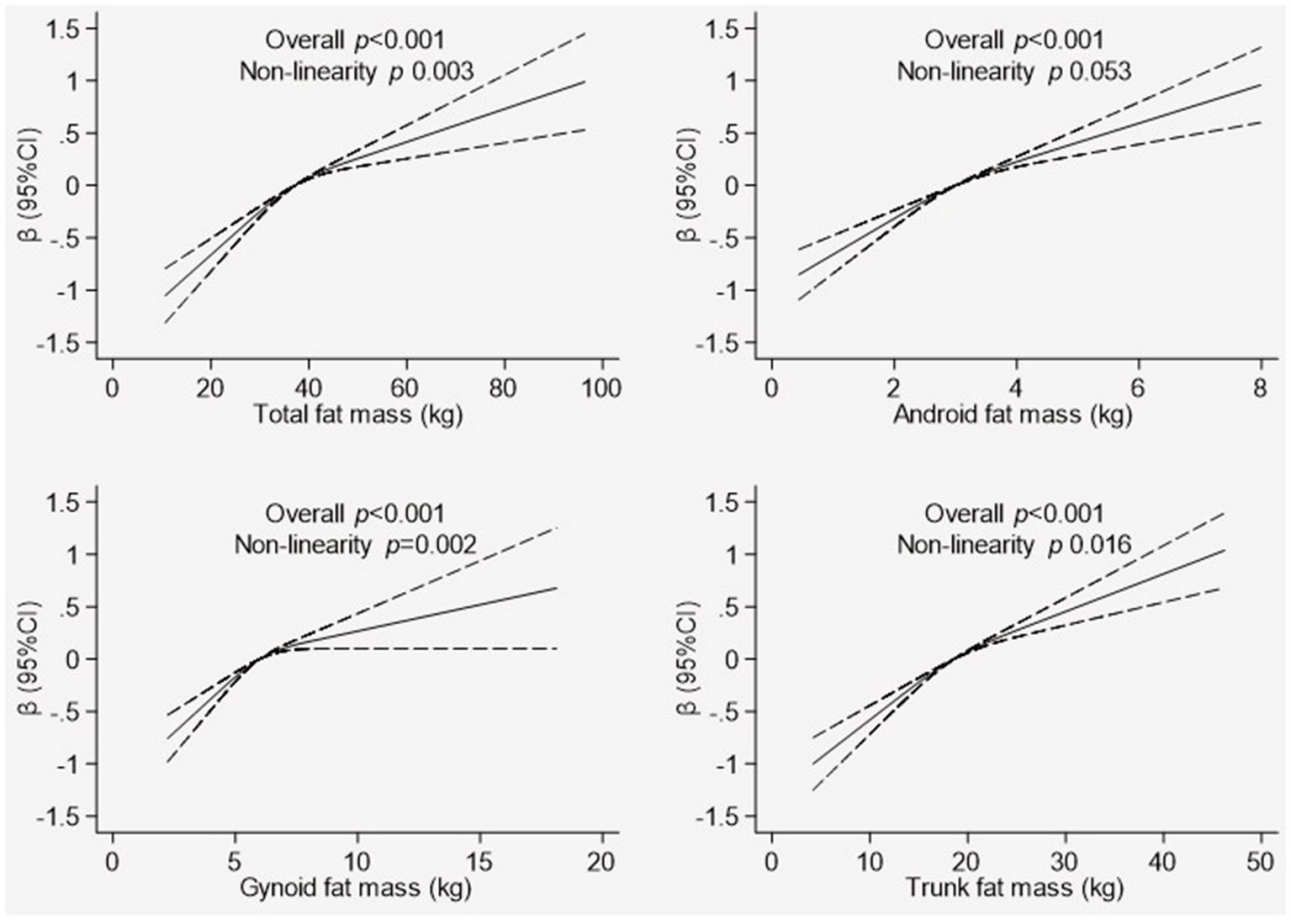
Association between whole body Tscore and body composition indicators. Graphical representation of non-linear association between total fat mass, android fat mass, gynoid fat mass, and trunk fat mass with T-score of total BMD derived using the restricted cubic spline method. The models were adjusted for are age, physical activity, supplement use, and smoking status. Dotted line represents the 95% confidence intervals.

The association between the T-score of the total BMD and components of fat distribution (total FM, android FM, gynoid FM, and trunk FM) are illustrated in Figure 1. The model was adjusted for age, physical activity, supplement use and smoking status. The fat distribution components exhibited a significant, non-linear relationship with the T-score (*p* < 0.001). In the graph of total FM, the line was not as steep after the total FM reached 40 kg. For android FM, the approximate threshold value was 4 kg and the increase in the T-score was not as steady for values above the threshold. The approximate threshold value for gynoid FM was 6 kg, and when the gynoid FM was below 6 kg, the T-score showed a small linear increase. In contrast, the association with T-score became weaker and the line approached a plateau for the gynoid FM >6 kg. In the trunk FM graph, the approximate threshold value was 20 kg, and the line was not as steep when the trunk FM was above 20 kg.

## Discussion

This population-based cross-sectional study evaluated the association between body composition and BMD indicators in Qatari women (≥18 years) based on recent data from the QBB that was obtained in August 2021. To the best of this research group's knowledge, this was the first study to demonstrate the relationship between fat distribution and BMD among Qatari women. The prevalence of obesity in this study population was higher (51.5%) compared with results from the Qatar health report 2014–2016, where the prevalence of obesity in women was 43.2% (10). This rate of increase is consistent with the fact that the prevalence of obesity in Qatar is increasing (18). Compared with FM, a strong positive association was found between LM and BMD at different sites (lumbar spine, femur, and FN), as well as with total BMD and the T-score of total BMD. The association between fat distribution variables and the T-score of total BMD resulted in a significant non-linear curve.

According to this study, those with obesity had a higher BMD, which was consistent with the study by Salamat et al. (15), where obese premenopausal and postmenopausal women (BMI ≥25 kg/m^2^) had higher BMD compared with those with normal weight (BMI <25 kg/m^2^). Similarly, other studies have reported that obesity was positively related to an increased bone mass (13, 14, 16). Qiao et al. (16) stated that a possible reason for the positive association of obesity with BMD was that high body weight contributes to a mechanical loading effect on bone, which enhances the activation of osteoblasts and the formation of bone. In contrast, Gameil et al. (18) showed that among obese Egyptian premenopausal women and those of normal weight, obesity was associated with an increased risk of low BMD. The study revealed a significant inverse relationship (*p* < 0.001) between obesity variables (e.g., BMI) and BMD indicators at different sites (e.g., z-score at lumbar spine). Additionally, Beck et al. (37) showed that although heavier individuals have higher hip BMD, women with the highest BMI reported more falls and fractures, and had lower measures of physical activity and function. Therefore, a critical analysis of region-specific BMD should be undertaken.

Age and BMI-adjusted partial correlation indicated that total LM had a significant positive correlation with BMD at different regions, and this agreed with the findings of several other studies (29, 38). A study by Xiao et al. (27) among women (20–95 years), reported that total LM had a greater protective effect on BMD in premenopausal women. They concluded that the effect of LM on BMD could be ascribed to the mechanical influence of muscle, which generates a positive effect on osteogenesis. The findings of this study also indicated that total LM was more positively associated with BMD indicators than the components of fat distribution. Similar findings were observed by Ilesanmi-Oyelere et al. (17) for postmenopausal women. However, Wang et al. (26), in a study of postmenopausal women, found that when adjusted for age, both FM and LM had positive associations with BMD at the lumbar spine, FN and hip regions. This difference in findings might be explained by the association of body composition with bone mass being significantly determined by the parameter of the bone analyzed (39). Additionally, both FM and LM have crucial effects on bone mass, based on the bone parameters used, the site of skeleton where measurements were taken and menopausal status (39).

This study also found that gynoid FM and total FM had a significant negative correlation with BMD at different regions. Similar findings were reported by Casale et al. (32), where TFM was negatively correlated to BMD among premenopausal women. Conversely, the results were inconsistent with those of Kapuš et al. (22) and Namwongprom et al. (25) among postmenopausal European and Thai women, respectively. This contradiction could be due to the differential effect of FM on bone among postmenopausal and premenopausal women. While this study did not categorize the participants according to their menopausal status, total FM might be a stronger indicator of BMD in postmenopausal rather than premenopausal subjects (38). However, the results of this research agree with those of Salamat et al. (15), which indicated that when BMI was considered as a covariate, the association of TFM with BMD became negative. This negative effect with increased TFM was attributed to the increased levels of cytokines that are proinflammatory. Similarly, Kim et al. (23) found that the association of TFM with BMD among Korean premenopausal women (≥20 years) in weight-adjusted models became negative, which shows that high FM could have a detrimental effect on bone, despite the beneficial effect of mechanical loading.

Furthermore, this study observed a non-linear relationship between the components of fat distribution and the total BMD indicator (T-score), where the slope was not as steep as the TFM increased. An increased TFM might not have a protective effect on bone. This finding was comparable with the conclusions of Shi et al. (31) in a study of Chinese postmenopausal women. They explained that TFM has a curved association with bone indicators, and a strong protective influence of TFM on bone occurs only in thin people. For overweight and obese individuals, increased TFM could be damaging to bone health.

Several potential mechanisms have been proposed to clarify the relationship between body composition parameters and bone mineral indicators. The LM could positively affect the bone through muscle contractions, along with the loading effect due to the force of gravity (38), and the advantageous hormonal effect of the skeletal musculature (40). For example, muscle fibers release an insulin-like growth factor 1 (IGF-1) which can trigger the growth of bone via receptors or the signaling mechanism of IGF (41). The effect of TFM on bone is probably more complex, because both bone and adipose cells originate from a pluripotent mesenchymal stem cell (42), and these cells can transform into both adipocytes and osteoblasts (42). The TFM possibly affects the bone positively via the weight-loading mechanism (32), and modulation of the endocrine pathway (38). Adipocytes release a lot of hormones, including leptin, insulin, and adiponectin, that impact bone metabolism (38). Additionally, the adipose tissue contains an aromatase enzyme that changes androgen to estrogen, leading to a high release of estrogen (38). These hormones have a protective effect on bone and results in bone development by initiating osteoblast differentiation and the prevention of resorption by osteoclast. In addition, estrogen enhances the synthesis of protein in muscles and the deposition of bone calcium, leading to an increase in BMD (43). On the other hand, increased adiposity causes inflammation and increased inflammation promotes bone resorption (44). Adipose tissue cells secrete a variety of proinflammatory cytokines, such as interleukin 6 (IL-6) and TNF-α (tumor necrosis factor-alpha). These factors activate bone resorption and inhibit bone formation by the increased stimulation of the receptor activator of nuclear factor-kB ligand (RANKL), which mediates osteoclast formation (45). Furthermore, excessive adiposity also leads to a decrease in the release of the adiponectin hormone which is associated with differentiation of osteoblasts (21). Therefore, excess adiposity causes a decline in BMD.

In diagnosing those with a low bone mass, this study highlighted the importance of considering the impact of fat distribution at different skeletal sites rather than relying on total BMD. This finding has clinical implications as it would be helpful in improving the diagnostic criteria for conditions associated with low bone mass. Future studies could analyse the effect of fat distribution on BMD at different skeletal sites and the variation in the results among obesity categories.

This study had both strengths and limitations. One of the limitations was that a cross-sectional study design prevented the determination of causal association between body composition indicators and BMD. Second, only the total BMD T-score was considered for determining the association of fat distribution with BMD; the BMD at different skeletal sites was not included. Additionally, the study population could have affected the results as they mostly comprised overweight and obese participants. There could also be confounding bias due to residual confounders that were not included in the analysis, such as sex-hormone status, menopausal status, and the levels of bone biomarkers. One of the strengths, however, was that the data obtained from the QBB included a large sample size with anthropometric measurements, body composition variables and bone mineral density indicators.

## Conclusion

This study illustrates that LM is a stronger predictor of BMD compared with FM. Additionally, it showed that FM might not have a strong protective effect on bone health as the association of FM with T-score was non-linear and there was not much increase in the T-score when FM increased. Further investigations are necessary to confirm the association between FM and BMD by considering BMD at different regions of the body, as total body BMD might not be a better indicator of bone mass.

## Data Availability Statement

The datasets presented in this article are not readily available due to the confidential nature of the material. However, they can be available on request to Qatar Biobank study management team. Requests to access the datasets should be directed to https://www.qatarbiobank.org.qa/.

## Ethics Statement

The studies involving human participants were reviewed and ethical approval was obtained from the Institutional Review Board of the Qatar Biobank (EX-2021-QF-QBB-RES-ACC-00040-0166) and carried out according to the Declaration of Helsinki. A written consent was obtained for all participants to share their data.

## Author Contributions

AK and MR conceived and designed the study. SL, YK, TT, and GA contributed to the original draft preparation. AK wrote the manuscript, has full access to all data, and is responsible for the results accuracy. MR and AA critically reviewed data analysis and contributed to the writing and editing of the manuscript. AK and ZS performed the statistical analysis. All authors have read and agreed to the published version of the manuscript.

## Conflict of Interest

The authors declare that the research was conducted in the absence of any commercial or financial relationships that could be construed as a potential conflict of interest.

## Publisher's Note

All claims expressed in this article are solely those of the authors and do not necessarily represent those of their affiliated organizations, or those of the publisher, the editors and the reviewers. Any product that may be evaluated in this article, or claim that may be made by its manufacturer, is not guaranteed or endorsed by the publisher.
